# Costs and Cost-Effectiveness at 12 and 24 Months of an Enhanced Web-Based Physical Activity Intervention for Latina Adults: Secondary Analysis of a Randomized Controlled Trial

**DOI:** 10.2196/76739

**Published:** 2025-11-25

**Authors:** Britta Larsen, Dori Pekmezi, Sheri J Hartman, Shira Dunsiger, Todd Gilmer, Erik Groessl, Bess Marcus

**Affiliations:** 1 Herbert Wertheim School of Public Health University of California, San Diego San Diego, CA United States; 2 Department of Health Behavior School of Public Health University of Alabama Birmingham United States; 3 Department of Behavioral and Social Sciences School of Public Health Brown University Providence, RI United States; 4 VA San Diego Healthcare System San Diego United States

**Keywords:** cost-effectiveness, physical activity, Latino health, health disparities, mobile health, mHealth

## Abstract

**Background:**

We previously established the efficacy and cost-effectiveness of a web-based physical activity (PA) intervention for Latina adults, which increased PA, but few participants met PA guidelines, and long-term maintenance was not examined. A new version with enhanced intervention features was found to outperform the original intervention in long-term guideline adherence.

**Objective:**

This study aimed to determine the costs and cost-effectiveness of the enhanced multitechnology PA intervention compared to the original web-based intervention in increasing minutes of activity and adherence to guidelines.

**Methods:**

Latina adults (N=195) were randomly assigned to receive a Spanish-language, individually tailored web-based PA intervention (original) or the same intervention with additional phone calls and interactive SMS text messaging (enhanced). PA was measured at baseline, 12 months (end of active intervention), and 24 months (end of tapered maintenance) using self-report (7-day PA recall interview) and ActiGraph accelerometers. Costs were estimated from a payer perspective and included all features needed to deliver the intervention, including staff, materials, and technology. Cost-effectiveness was calculated as the cost per additional minute of PA added over the intervention and the incremental cost-effectiveness ratios of each additional person meeting guidelines.

**Results:**

At 12 months, the costs of delivering the interventions were US $16 per person per month in the enhanced arm and US $13 per person per month in the original arm. These costs decreased to US $14 and US $8 at 24 months, respectively. At 12 months, each additional minute of self-reported activity in the enhanced group cost US $0.09 compared to US $0.11 in the original group (US $0.19 vs US $0.16 for ActiGraph), with incremental costs of US $0.05 per additional minute in the enhanced group beyond the original group. At the end of the maintenance period (24 mo), costs per additional minute decreased to US $0.06 and US $0.05 (US $0.12 vs US $0.10 for ActiGraph), with incremental costs of US $0.08 per additional minute in the enhanced group (US $0.20 for ActiGraph). Costs of meeting PA guidelines at 12 months were US $705 in the enhanced group compared to US $503 in the original group and increased to US $812 and US $601 at 24 months, respectively. The incremental cost-effectiveness ratio for meeting guidelines at 24 months was US $1837 (95% CI US $730.89-US $2673.89) per additional person in the enhanced group compared to the original group.

**Conclusions:**

The enhanced intervention was more expensive but yielded better long-term maintenance of activity, costing US $1837 per extra person meeting guidelines beyond those in the original group. Both conditions were low cost relative to other medical interventions. The enhanced intervention may be preferable in populations at high risk, where more investment in meeting guidelines could yield more cost savings.

**Trial Registration:**

ClinicalTrials.gov NCT03491592; https://clinicaltrials.gov/study/NCT03491592

**International Registered Report Identifier (IRRID):**

RR2-10.1186/s13063-022-06575-4

## Introduction

There is a critical need for scalable, cost-effective interventions to address high rates of physical inactivity and related chronic diseases, especially in underserved populations considered to be at risk. In fact, the World Health Organization estimates that further inaction could lead to 500 million new global cases of preventable noncommunicable diseases, with associated direct health care costs of US $520 billion, by 2030 [1]. Internet-based physical activity (PA) interventions have great potential for widespread dissemination and have already been shown to improve PA levels [2-4]. However, most of this work was conducted in the general population and not extended to those most at risk, including underrepresented minority populations.

Hispanic adults report the highest rates of inactivity outside of work (32.1% vs 23% for non-Hispanic White individuals) and experience disproportionate rates of associated conditions (eg, obesity and diabetes) [5,6]. In fact, 79% of Latinas are overweight or obese, compared to 64% of non-Hispanic White women [5]. Thus, in the Pasos Hacia La Salud study, we tested a web-based, PA intervention in Spanish with 205 Latinas and found significantly greater increase in weekly minutes of moderate-to-vigorous PA (MVPA) compared to a control condition [7,8]. Moreover, the costs associated with providing the intervention were low at 6 months (US $17 per person per month) and further decreased (US $12 per person per month) at 12 months [9]. While the Pasos program was shown to be effective and low cost, most intervention participants (69%) did not meet national PA guidelines at 6 months [7,8,10], and longer-term maintenance was not evaluated.

To help sedentary Latinas achieve and maintain health-enhancing levels of PA, the intervention was refined by adding SMS text messages and further targeting key social cognitive theory [11] variables (eg, self-efficacy, enjoyment, and social support). Subsequently, in the Pasos Hacia La Salud II study, the new technology- and theory-enhanced version was tested and compared with the original Pasos Hacia La Salud intervention in a randomized controlled trial (N=205) [12]. There were no significant between-group differences in PA at 6 or 12 months; however, the enhanced intervention arm had higher PA levels than the original intervention arm at 18 and 24 months [13].

While both programs have been shown to be beneficial, the enhanced intervention appears to have an advantage in terms of long-term behavior change. However, these enhancements (eg, SMS text messages, discussion board, and additional staff contacts) likely come at additional expense (compared to the original arm). An evaluation of intervention costs is needed to determine which program is feasible and the best fit for the clinical and community setting and resources. Thus, to inform future dissemination and implementation efforts, this study examined additional costs associated with intervention enhancements and how they influence cost-effectiveness.

## Methods

### Overview

Pasos Hacia La Salud II was a fully powered randomized trial of 2 PA interventions: the original web-based PA intervention (original) and an enhanced arm that included additional elements to support increasing activity (enhanced). Participants were adult Latinas aged 18 to 65 years who were underactive (engaging in <60 min of MVPA per week), could read and write in Spanish, had access to technology to use the study website and receive SMS text messages, and were healthy enough for unsupervised exercise. A full description of the study protocol, measures, and participants has been published previously [13,14].

### Intervention Description

In the original arm of the web-based intervention, participants attended a baseline session led by trained bilingual interventionists who explained MVPA and helped participants set incremental goals. Participants also completed goal-setting sessions over the phone at 1 and 9 months and at follow-up visits (6 and 12 mo). Participants were encouraged to accumulate MVPA on most days of the week and encouraged to achieve the goal of 150 minutes of MVPA by 6 months. All participants were given a pedometer (Accusplit Inc) and were encouraged to use it to self-monitor daily activity.

The interventionist also oriented participants to the study website, which participants could access throughout the intervention period (24 months). The website included tools to help participants increase their MVPA, such as self-monitoring, goal setting, social support, instruction about how to be physically active, problem solving, motivational stage-matched PA manuals, and individually tailored feedback. Further details about the tailored intervention content are described elsewhere [14]. Participants were encouraged to regularly access the website, log their PA on the website, and receive a tip of the week. Participants were also prompted to complete online surveys to customize their intervention content. Most web-based content was automated and did not require staff time. However, staff regularly checked the message boards to answer questions as necessary and were available to help participants with any technology issues.

In addition to the intervention components described earlier, the enhanced intervention included (1) SMS text messages; (2) additional phone calls at 2, 3, 15, and 21 months; (3) additional in-person visits at 18 and 24 months; and (4) additional data-driven content and website features that encouraged user participation. Participants were also provided with nonmonetary incentives for their website use. Participants earned points by logging into the website and using certain features, such as the goal-setting calendar. Once participants earned a given number of points, they were sent prizes, such as water bottles, phone cases, and T-shirts.

To further enhance social support for PA, a discussion board was used to facilitate meetups among participants. Research staff posted details about free and low-cost PA events in the area so that participants could meet, discuss coordinating attendance with others, overcome barriers to PA, and motivate each other to attend these events.

### Ethical Considerations

The study was reviewed and approved by the Brown University Human Research Protections Program (1708001868), and all participants provided written informed consent in the language of their choosing (English or Spanish). To protect confidentiality, all data were deidentified for storage and analysis. To thank them for their time, participants received compensation for participating in study activities. Participants received US $25 for completing each of their assessment visits (at baseline and 6, 12, 18, and 24 months) with an additional US $50 bonus at 24 months if they completed all visits. In addition, all participants received incentives for filling out monthly questionnaires (US $10 per month) to generate the tailored website content. Participants also received reimbursement costs related to travel and childcare costs. Participants in the enhanced arm also received prizes for engaging with website content (as mentioned earlier). This trial has been registered at ClinicalTrials.gov (NCT03491592),

### PA Measures

The main outcomes used to determine cost-effectiveness were the change in minutes of MVPA from baseline to 12 and 24 months, as measured by ActiGraph accelerometers and self-report, and the percentage of participants meeting national guidelines at 12 and 24 months. Self-reported activity was measured using the 7-day PA recall (PAR) interview, an interviewer-administered measure that has shown good reliability and validity in adult populations, including in Spanish populations [15-17]. Adherence to national guidelines was defined as engaging in ≥150 minutes per week of MVPA, as measured by the 7-day PAR [18].

### Costs

#### Overview

Cost calculations followed the model used in our previous publications [9]. Costs were calculated from a payer perspective to estimate the amount it would cost to deliver the intervention in a clinical or community setting. This included all costs associated with personnel, materials, and use and maintenance of technology to deliver the intervention, sourced from actual costs incurred during the trial.

Costs associated with research, such as recruitment, baseline and follow-up measurement visits, obtaining consent, or compensation for research participation, were not included. We also did not include costs associated with intervention development. Thus, both research and development costs are not relevant to the future implementation of the existing intervention in community or clinical settings. Excluding these costs is consistent with published guidelines on cost-effectiveness analyses [19].

#### Personnel Time

Staff time logs were used to determine the time needed to complete each activity associated with intervention delivery. This included training time (for both the trainer and the trainee); time to compile study materials; time for study visits and phone calls (depending on study condition); and time for study maintenance activities, such as checking the online message board, resending pedometers to participants who lost them, calling the website developer with technical questions, or (in the enhanced group) offering SMS text message support. Study maintenance activities were conducted during a set time each week, rather than calculated as a per-participant activity.

Costs for personnel were calculated by determining the total time needed to deliver the intervention per participant in each condition and multiplying by actual staff salary rates, including benefits. Staff delivering the intervention were entry-level research staff with an undergraduate degree (earning a yearly salary of US $54,080 plus 31% in benefits, for a total hourly cost of US $34.05). Training staff were master’s-level behavioral scientists (earning a yearly salary of US $68,640, plus 31% in benefits, resulting in a total hourly cost of US $43.20). Overhead was estimated as an additional 10% of all personnel costs to account for the use of shared space.

#### Technology

Web hosting was US $75 per month for both study arms. The automated SMS text messaging was supported through the study website and thus did not incur any additional costs.

#### Materials

Costs for materials were based on actual costs incurred during intervention delivery. All participants in both study arms received an Acusplit pedometer to record their daily steps for US $20 each. Participants in both arms also received a study binder at their baseline visit with information about exercise, tip sheets, and logs for using their pedometers. The total cost for the binders and printed materials was US $4.95 for each participant.

Total cost for prizes in the enhanced group was calculated based on the actual cost of the items and the number of each item that was distributed. As not all participants earned all prizes, we calculated an average cost per participant for prizes to allow for an estimate of the actual cost of delivering prizes at varying rates based on participant engagement. Prize costs ranged from US $1.11 each for water bottles to US $12.50 for a yoga mat. Total cost for prizes in the enhanced arm was US $2038.

A computer was also purchased for the study staff to conduct baseline study visits, check the study website message boards, etc. The cost of the computer was US $1200.

### Analysis

To allow for comparison, we used the same analysis approach as in the parent study [9]. Costs were calculated from a payer perspective to estimate the cost of implementing the intervention in a community or clinical setting. Total costs were calculated separately for each study arm and included the total of personnel time (including benefits and use of shared space), materials, and web hosting. Total costs for each arm were divided by the number of participants in each study arm to calculate the cost per person to deliver each intervention. Costs were calculated in US dollars for 2024.

Cost-effectiveness was calculated as the cost per additional minute of MVPA per person over the course of the study. Total increase in minutes was calculated using linear interpolation of minutes across measurement time points (baseline and 6, 12, 18, and 24 months) and subtracting baseline minutes. Main outcomes for costs and cost-effectiveness were calculated at 12 months (main study outcome) and cumulatively at 24 months. The cost of intervention delivery per person was divided by the cumulative increased minutes per person over the course of the study to estimate the cost per additional minute per person. This was done both for self-reported activity (7-day PAR) and objectively measured activity (ActiGraph accelerometer). Similarly, the cost of each intervention was divided by the number of participants in that group who met national guidelines at each time point to estimate the cost of moving 1 person from being inactive to successfully meeting guidelines for activity at 12 and 24 months.

Incremental cost-effectiveness ratios (ICERs) were calculated to determine the additional cost per minute of MVPA in the enhanced group beyond that achieved by the original group, as well as the cost per additional individual meeting guidelines in the enhanced group compared with the original group. ICERs were calculated by dividing the difference in the costs between the 2 study arms by the difference in cumulative minutes between the 2 arms and, separately, by the difference in the number of people meeting guidelines. For ICERs for guidelines, CIs were computed using the nonparametric bootstrap with 1000 replications.

## Results

### Overview

Participants (N=195) had a mean age of 43.3 (SD 10.29) years and were primarily of Dominican (80/195, 41%) and Colombian (33/195, 16.9%) descent. Approximately half of the participants (92/195, 47.2%) had a high school education or less. A full description of baseline characteristics and a CONSORT (Consolidated Standards of Reporting Trials) diagram have been published previously [13].

### PA Changes

As published previously [13], participants in the enhanced group (103/195, 52.8%) increased self-reported weekly MVPA from 57.9 minutes at baseline to 115 minutes at 6 months, 106.8 minutes at 12 months, and 135.3 minutes at 24 months, while the original group (92/195, 47.2%) increased self-reported weekly MVPA from 55.2 minutes at baseline to 88 minutes at 6 months, 111.7 minutes at 12 months, and 93.9 minutes at 24 months [13]. ActiGraph-measured weekly MVPA in the enhanced group changed from 19.7 minutes at baseline to 47 minutes at 6 months, 44.5 minutes at 12 months, and 47.4 minutes at 24 months. In the original group, ActiGraph-measured weekly MVPA changed from 20.6 minutes at baseline to 43 minutes at 6 months, 55.9 minutes at 12 months, and 31.2 minutes at 24 months.

None of the participants met national MVPA guidelines at baseline. At 12 months, 28.2% (29/103) and 29% (27/92) of participants met guidelines according to self-report in the enhanced and original conditions, respectively. At 24 months, this increased to 36.9% (38/103) and 31% (29/92) in the enhanced and original groups, respectively.

### Costs

Total costs associated with delivering the interventions are shown in Table 1. Total cost of delivering the original intervention at 12 months was US $15,741, or US $171 per person (US $14 per person per month). The enhanced intervention cost US $20,435 to deliver over the first 12 months, or US $198 per person (US $16 per person per month).

**Table 1:** Costs of delivering the enhanced and original interventions.

Costs			Original intervention (n=92)					Enhanced intervention (n=103)		
			12 mo		24 mo (cumulative)		12 mo			24 mo (cumulative)
**Personnel, US $**										
	Training	510		510		510			510	
	Intervention delivery	10,387		10,723		13,495			21,845	
**Website, US $**										
	Hosting	900		1800		900			1800	
	Technical support	449		899		449			899	
**Materials, US $**										
	Computer	1200		1200		1200			1200	
	Pedometers	1840		1840		2060			2060	
	Paper, binders, etc	455		455		510			510	
	Prizes	—^a^		—		1311			2038	
Total costs, US $			15,741		17,428		20,435			30,862
Average cost per participant, US $			171		189		198			300
Average cost per participant per month, US $			14		8		16			13

^a^Not applicable.

At 24 months, cumulative costs for the original intervention increased slightly to US $17,428, or US $189 per person (US $8 per person per month). However, the cumulative costs for the enhanced intervention increased to US $30,862 at 24 months, or US $300 per person (US $12 per person per month). The largest source of the difference was personnel cost for intervention delivery between 12 and 24 months (Tables 1 and 2).

**Table 2:** Staff time needed for intervention activities.

Time			Original intervention (n=92)				Enhanced intervention (n=103)		
			12 mo		24 mo (cumulative)		12 mo		24 mo (cumulative)
**Training, min**									
	Trainee	360		360		360		360	
	Trainer	360		360		360		360	
**Intervention activities per person, min**									
	Assembling materials	5		5		5		5	
	Baseline goal setting	60		60		70		70	
	1-wk call	10		10		15		15	
	1-mo call	25		25		30		30	
	2-mo call	—^a^		—		10		10	
	3-mo call	—		—		10		10	
	6-mo goal setting	25		25		30		30	
	9-mo call	25		25		30		30	
	12-mo goal setting	—		25		—		30	
	15-mo call	—		—		—		30	
	21-mo call	—		—		—		30	
	18-mo goal setting	—		—		—		30	
**Study maintenance activities (not per person), min**									
	Technical support	720		1440		720		1440	
	Message board and injury check	360		720		360		720	
	Resending pedometers	180		360		180		360	
	SMS text message support	—		—		480		960	

^a^Not applicable.

### Cost-Effectiveness

Cost per increased minute of activity is shown in Table 3. For self-reported activity, each additional minute gained over 12 months in the enhanced group cost US $0.09, compared with US $0.11 per minute in the original group. Incremental costs of increased minutes in the enhanced group were US $0.05 per minute beyond those reported by the original group.

As increases in MVPA measured by the ActiGraph were smaller, these also cost more, with each increased minute costing US $0.19 in the enhanced group and US $0.16 in the original group. As increases in the original group were larger than those in the enhanced group, ICERs could not be calculated.

Costs for cumulative increases over the 24-month period were lower. Each self-reported increased minute cost US $0.06 in the enhanced group and US $0.05 in the original group. Incremental increases in the enhanced group beyond the original group were US $0.08 per additional minute of self-reported MVPA. ActiGraph-recorded minutes over the 24-month period were US $0.12 in the enhanced group compared to US $0.10 in the original group, with incremental minutes costing US $0.20 each in the enhanced group beyond that in the original group.

Cost per person meeting guidelines was normalized per 100 people in each arm. This was higher in the enhanced arm at 12 months (US $705) than in the original arm (US $503). These rose to US $812 and US $601 at 24 months, respectively. ICERs at 24 months were US $1837 (95% CI US $730.89-US $2673.89) per additional person meeting guidelines in the enhanced arm beyond those in the original arm.

**Table 3:** Costs of increases in physical activity.

			Original group				Enhanced group		
			7-d PAR^a^		ActiGraph		7-d PAR		ActiGraph
**Total increase in MVPA^b^ per person, min**									
	Baseline to 12 mo	1584		1038		2120		1038	
	Total 24 mo	3491		1921		4935		1921	
**Costs per minute increase in MVPA,** **US $**									
	Baseline to 12 mo	0.11		0.16		0.09		0.16	
	Total 24 mo	0.05		0.10		0.06		0.10	
**Incremental cost per minute of increase in** **MVPA,** **US** **$**									
	Baseline to 12 mo	—^c^		—		0.05		—	
	Total 24 mo	—		—		0.08		—	
**Costs per person meeting guidelines, US $**									
	12 mo	503		—		705		—	
	24 mo	601		—		812		—	
**Incremental cost per person meeting guidelines, US $**									
	12 mo	—		—		N/A^d^		—	
	24 mo	—		—		1837 (730.89-2673.89)		—	

^a^PAR: physical activity recall.

^b^MVPA: moderate-to-vigorous physical activity.

^c^Not applicable.

^d^Not available. As the original arm outperformed the enhanced arm in these metrics, it was not possible to calculate the incremental cost-effectiveness ratios.

## Discussion

### Principal Findings

Analyses showed that the technology- and theory-enhanced PA intervention for Latinas was more costly than the original intervention but still markedly less costly than most medical interventions. The enhanced intervention cost US $300 per person for a 24-month program, or approximately US $12 per person per month. The original intervention cost US $189 per person, or approximately US $8 per person per month. The largest expense by far was personnel time, which accounted for approximately 72% and 64% of costs in the enhanced and original groups, respectively. Most of the increased cost in the enhanced intervention was attributable to additional personnel time for making additional monthly calls and providing SMS text message support. The prizes also contributed to higher costs in the enhanced group.

Costs and activity gains were similar throughout the first year, thus cost-effectiveness was also similar, with minutes gained in the enhanced group costing US $0.09 for each participant compared to US $0.11 for each participant in the original group (US $0.19 and US $0.16 by ActiGraph, respectively). During the second year in the program, the enhanced group continued to increase their MVPA, while gains in the original group declined. However, the original group had few costs incurred in the second year, while the enhanced group continued to deliver maintenance doses of intervention. Cost-effectiveness thus remained similar over the full 24 months, with each additional minute costing just US $0.05 in the original group and US $0.06 in the enhanced group (US $0.10 and US $0.12 by ActiGraph, respectively). ICERs showed that, beyond the cost of the original intervention, each additional minute in the enhanced intervention cost just US $0.08 (US $0.20 for ActiGraph-measured minutes). While more individuals in the enhanced group met guidelines at 24 months, the cost of meeting guidelines was also higher, costing US $812 per person compared to US $601 in the original arm.

While absolute minutes of PA varied between self-report and objective measures, as is commonly seen between these 2 measures [20,21], the overall pattern of results between the 2 were similar. While accelerometry is considered the gold standard, there is a large body of research showing the benefits associated with self-reported activity, which largely informed the development of national guidelines [22,23]. Self-reported MVPA allows for subjective interpretation of intensity, which may not align with universally applied cut points for accelerometers, particularly for participants who are overweight, obese, or inactive. Both the 7-day PAR and ActiGraph showed sustained increases in activity in the enhanced group over the 24-month study period, corroborating better maintenance of activity gains.

### Comparison With Prior Work

These results suggest that paying for more intervention yields commensurate increases in activity. Given the enormous benefits of PA [18,24-26], particularly if it is maintained over time [26,27], the additional cost of more intensive interventions that yield greater increase in PA over time is likely preferable for implementation sites when feasible. Although we could not find other studies that framed the incremental cost of an intervention in terms of additional people meeting PA guidelines, meeting PA guidelines is recognized as an important research metric [23]. One health economics study concluded that PA interventions that cost less than US $2900 over 2 years to help persons meet PA guidelines can be considered cost-effective [28]. Our finding that the enhanced group spent US $1837 per additional person meeting guidelines compared to the original group at 24 months is well below this threshold, and therefore, the intervention can be considered cost-effective.

This amount also seems a relatively small price to pay compared to the cost of managing chronic diseases associated with inactivity. The cost of managing diabetes, for example, was US $237 billion in the United States in 2017, with insulin alone costing approximately US $5000 per user annually [29,30]. An analysis of health care use in Australia found that individuals meeting activity guidelines were about one-third less likely to visit the emergency room or be hospitalized, half as likely to use outpatient services, and incurred approximately Aus $1400 (US $920) less in annual health care costs [31]. Multiple studies have shown that PA programs are not only low cost but ultimately cost saving, saving considerably more in health care costs than the programs cost [32,33]. One evaluation of a PA intervention for older community-dwelling adults found that, for those not initially meeting activity guidelines, the program saved US $143 to US $164 per participant over 6 months beyond the cost of the intervention [33]. Paying for PA programs can therefore be seen as an investment not only in the health and well-being of the participants but also in health care, particularly for populations at high risk. Given the higher costs and higher yields of the enhanced intervention, implementation of the enhanced version may be most appropriate with clinical populations managing chronic disease. As the original intervention still yielded substantial increases in activity, it may be more appropriate for community settings focusing on prevention and overall well-being.

Compared to the parent study, the original intervention in this study was slightly more expensive, costing US $14 per person per month at 12 months versus US $12; this was due to increased personnel costs due to wage increases [9]. Cost to implement the interventions will thus be largely dependent on staff salaries, which could vary broadly. Clinical sites could deliver the intervention via medical assistants or nursing staff, which would likely increase costs; conversely, using volunteers or automating components to reduce staff time could substantially lower costs.

### Limitations

This study has several limitations. Cost and cost-effectiveness analyses were based on aggregated costs data, not individual cost data, thus results should be interpreted as overall estimates for delivering the intervention rather than individual effectiveness. The costs were also limited to a payer perspective and did not include health care or societal costs or quality-adjusted life years. However, these approaches allow for direct comparability with the previous study, which used the same analytic approach. Moreover, some costs, such as overhead for shared space, could only be estimated and would vary considerably based on the implementation site. We were unable to determine the effectiveness of each intervention component; therefore, it was not possible to determine the added cost-effectiveness of individual intervention components. Key differences between the conditions were the SMS text messaging and the additional calls and in-person visits. The cost of these components varied greatly, with SMS text messaging being almost free and highly scalable and calls and visits being costly and having limited scalability. Future trials using multiphase optimization strategy (MOST) designs should be used to identify the most effective components to optimize the cost-effectiveness of the intervention.

Finally, the study findings may not generalize to other populations or settings. The study population was primarily of Caribbean descent and l resided in a small geographic region of the United States. The parent study was carried out with Latinas in California who were predominantly of Mexican descent and reported being markedly less active at baseline but who showed similar increases in activity. Future research with other populations and settings would elucidate how generalizable things findings are.

The study has several strengths. Data were taken from a randomized trial with rigorous methodology, including multiple validated measures of PA. The trial focused on an underserved population considered to be at high risk and included long-term follow-up. Costs were based on current market prices, including published pay scales and benefits for staff. We were also able to directly compare costs and cost-effectiveness to those in the previous parent randomized controlled trial.

### Conclusions

Findings highlight that additional intervention components, particularly those necessitating more staff time, yield higher intervention costs; however, they may also lead to greater increases in PA. In this study, these differences were most apparent after long-term follow-up. This could suggest that greater investment may be most appropriate in individuals who would benefit the most from long-term adherence to activity guidelines, such as those at high risk for, or managing, chronic diseases that respond to lifestyle changes. As staff time was by far the most costly component, PA interventions may become more cost-effective and more widely disseminated, as they rely more on broad reach technology. Future research should investigate whether relying fully on automated technology without face-to-face intervention components yields similar long-term effectiveness.

## Abbreviations

CONSORT: Consolidated Standards of Reporting Trials
ICER: incremental cost-effectiveness ratio
MOST: multiphase optimization strategy
MVPA: moderate-to-vigorous physical activity
PA: physical activity
PAR: physical activity recall
